# Mutant Kras-induced upregulation of CD24 enhances prostate cancer stemness and bone metastasis

**DOI:** 10.1038/s41388-018-0575-7

**Published:** 2018-11-22

**Authors:** Ching-Chieh Weng, Pei-Ya Ding, Yu-Hsuan Liu, John R. Hawse, Malayannan Subramaniam, Chia-Chen Wu, Yu-Chun Lin, Chiao-Yun Chen, Wen-Chun Hung, Kuang-Hung Cheng

**Affiliations:** 1 Institute of Biomedical Sciences, National Sun Yat-Sen University, Kaohsiung, 804 Taiwan; 2 Department of Biochemistry and Molecular Biology, Mayo Clinic, Rochester, MN 55905 USA; 3 Department of Medical Imaging, Kaohsiung Medical University Hospital, Kaohsiung, Taiwan; 4 National Institute of Cancer Research, National Health Research Institutes, Tainan, Taiwan; 5 Department of Medical Laboratory Science and Biotechnology, Kaohsiung Medical University, Kaohsiung, Taiwan

**Keywords:** Bone metastases, Prostate cancer, Cancer stem cells, Cancer models

## Abstract

Prostate cancer (PCA), one of the most common malignant tumors in men, is the second leading cause of cancer deaths in males worldwide. We report here that PCA models harboring conditional LSL/Kras^G12D^ or BRAF^F-V600E^ allele with prostate-specific abrogated p53 function recapitulate human PCA precursor lesions, histopathology, and clinical behaviors. We found that the development of reprogrammed EMT-like phenotypes and skeleton metastatic behavior requires concurrent activated Kras and p53 depletion in PCA. Microarray analyses of primary PCA cells derived from these models identified several cancer stemness genes including CD24, EpCAM, and CD133 upregulated by KRAS^G12D^. Among these stemness markers, we identified CD24 as a key driver of tumorigenesis and metastasis in vivo. These data demonstrate that specific factors involved in cancer stemness are critical for metastatic conversion of PCA and may be ideal targets for therapeutic intervention.

## Introduction

Genetic alterations predisposing to enhance prostate carcinoma (PCA) metastatic phenotype with major negative consequences for patient survival have not been fully elucidated [1–7]. Genes mutated with high frequency in PCA include PTEN (7%), P53 (14%), KRAS (7%), EGFR (3%), and BRAF (3%) [8–13]. Previous studies have elucidated that activated RAS protein directly interacts with at least three distinct effectors, RAF protein-Ser/Thr kinase, phosphoinositide 3-kinase (PI3K), and GDP/GTP Ral exchange factor, triggering downstream signaling responses including the Raf/MEK/Erk, PI3K/AKT, and Ral pathways [14, 15]. RAF and PI3K pathways are critical for tumorigenesis in many human tumors, synergistically regulating cell cycle progression, c-Myc protein stabilization and mTOR activation [16, 17]. Since genetic and pharmaceutical interference with PI3K activity can prevent ERK1 activation, cellular responses to RAS activation constitute a branching network of interacting PI3K/AKT and RAF/ERK pathways rather than independent signaling pathways [18, 19].

Some PCAs harbor mutations in the BRAF gene without KRAS mutations. Raf kinases can directly activate and phosphorylate MAP (mitogen-activated protein) kinase/ERK (extracellular signal-regulated kinase) kinases (MEK) to activate ERK. A single amino acid mutation can provoke BRAF activation [10]. The commonest BRAF mutation is a valine to glutamic acid substitution at position 600 (V600E), found in 90% of cancers, initiating ERK signaling by activating RAF-MEK-ERK signaling [11, 20]. Additionally, the P53 tumor suppressor gene, mutated in over 40% of PCA samples, contributes to genetic instability, aneuploidy, cytogenetic rearrangements and aggressive PCA [9, 21–23].

Recent studies established PTEN’s role in initiating PCA and identified p53 as an important constraint to progression [24]. The PB-Cre transgene directs Cre recombinase to the prostate from postnatal day 8.5 to abrogate LSL/KrasG12D, PTEN and p53 function [25]. One is lack of animal models that faithfully recapitulate PCA bone metastasis. In this study, we developed two novel genetic mouse models of PCA development and progression, a BRAF-driven PCA model and a Kras^G12D^ PCA mouse model, to determine how alterations in these genes contribute to signaling pathway activation, tumor invasion, and metastasis. The Kras^G12D^/p53 loss PCA model strikingly parallels human bone metastatic PCA. Kras^G12D^/p53loss induces cancer stem cell traits, with increased CD24, EpCAM, and CD133 expression as seen in advanced metastatic PCA. Activated CD24 induces Wnt signaling to induce cancer stemness in our mouse model, suggesting CD24/P-selectin inhibitor as a novel chemotherapy agent for metastatic PCA.

## Results

### Braf^V600E^-induced prostate carcinoma in conditional p53-deficient mice

**Fig. 1:**
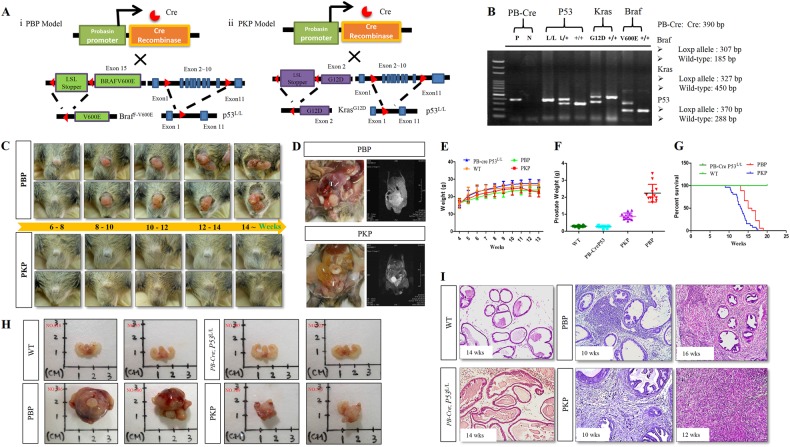
Activation of mutant Kras^G12D^ or BRAF^V600E^ synergizes with p53 deficiency to induce invasive PCA in mice. **a** Schematic comparison of panel i PBP and panel ii PKP PCA models with Probsin (PB) Cre-mediated activation of mutant BRAF^V600E^ or Kras^G12D^ and deleted P53 expression in mouse prostate. **b** PCR genotyping confirmed wild type and BRAF^F-V600E^, Kras^G12D^, and p53 floxed conditional homozygous or heterozygous alleles from the tail DNA of PKP and PBP offspring. **c** Morphological changes of external genitalia in mice of indicated genotypes. **d** MRI images of prostate tumors (T) from PBP and PKP mice. **e** Body weights of PB-Cre, PB-Cre;P53^L/L^, PBP, and PKP mice were evaluated every 3 days from birth to 4 weeks and compared to wild type. Data are mean ± SD, *n* > 6. **f** Prostate and prostate tumor wet weight of wild type, PB-CreP53^L/L^, PBP, and PKP 12-week-old mice (mean ± s.e.m.). ^*^P < 0.05, compared with wild type. **g** Kaplan–Meyer curve showing significantly reduced survival of PBP and PKP mice compared to wild type. *P* < 0.01. **h** Whole-prostate gross anatomy from mice of indicated genotypes. **i** H&E stained prostate sections of wild type and mutant mice of indicated genotypes. Magnification ×100

BRAF activating mutations were detected in 10% and inactivating mutations of tumor suppressor gene P53 are reported in 40% of human PCA [9, 11, 26]. To investigate whether deregulation of these genes induces PCA in mice, we crossed BRAF^F-V600E^ conditional and P53 conditional mutant mice with probasin (PB)-Cre transgenic mice. The modified probasin promoter drives postnatal expression of Cre recombinase in prostate epithelium, resulting in mutant Braf^V600E^ expression and excision of p53 (Fig. 1a–i). Polymerase chain reaction (PCR) genotyping confirmed the BRAF mutant allele and P53 deletion (Fig. 1b). Figure 1c, d shows the gross appearance and pathology of prostate lesions in macroscopic at 6, 8, 10, 12, and 14 weeks and MRI analyses of the PB-Cre;BRAF^V600E^;p53^L/L^ (PBP) mice. Figure 1e, f shows body and prostate tumor weights of different genotypes. Figure 1g shows cumulative tumor-free survival curves of mutant mice and controls. PBP mice develop multifocal and malignant PCA by 8 weeks of age with 100% penetrance and survive up to 16–17 weeks (*n* = 24 mice; Fig. 1g). PBP mice showed outward growth from the external genital organs forming large, irregular, highly vascular, and firm tumor masses (Fig. 1c, d). PCA from PBP mice was morphologically similar to human PCA with occasional foci of poorly differentiated carcinoma. In comparison, PB-Cre; BRAF^V600E^ and PB-Cre;p53^L/L^ mice (up to age 26 weeks) never developed PCA, indicating alterations to both genes are needed for tumor formation (Fig. 1h, i; Supplementary Fig. S1).

### Mutant Kras^G12D^ activation coupled with loss of p53 synergistically induces bone metastatic PCA

KRAS point mutations in exons 12 and 13 appear in 4–8% of human PCA [27]. To compare the effect of Braf^V600E^ and Kras^G12D^ on prostate tumorigenesis, we crossed PB-Cre Kras^G12D^ mice with p53^loxp/loxp^ mice to generate PB-Cre Kras^G12D^ p53^L/L^ (PKP) compound mice (Fig. 1a—panel ii, b). No evidence of PIN or PCA appeared at 30 weeks in aged PB-Cre Kras^G12D^ mice (*n* = 6) (Supplementary Fig. S1). PKP mice at the same age exhibited accelerated PCA development (*P* < 0.01) (Fig. 1c, d). Body and prostate tumor weights are shown in Fig. 1e, f. Interestingly, we observed the PBP tumors are larger than PKP tumor in our models. PKP mice started succumbing to PCA at 6 weeks and all died by 12–13 weeks (Fig. 1g). Noninvasive MRI longitudinally compared PCA abdominal metastasis in PKP and PBP mice (Fig. 1d). Figure 1h compares biopsy and macroscopic PCA in PKP and PBP mice. As early as 6 weeks PKP mice developed PCA with 100% penetrance (*n* = 25) by hematoxylin and eosin (H&E) histology (Fig. 1i).

**Fig. 2:**
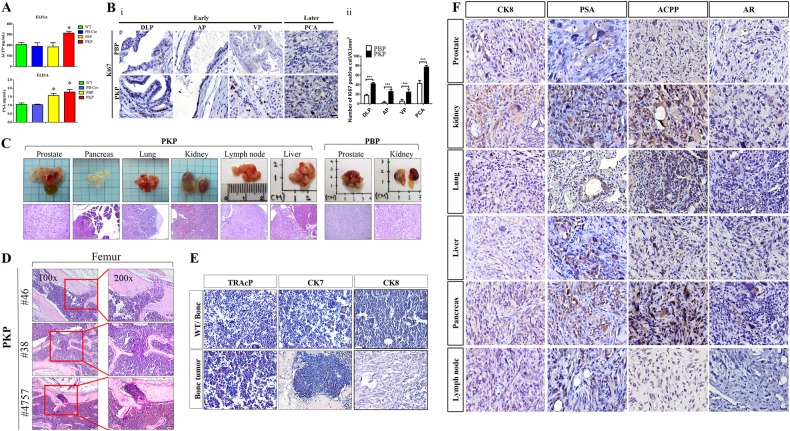
Molecular and histological characterization of metastasis in PBP and PKP mice. **a** ELISA assays of ACPP and PSA in wild type, PKP and PBP blood. **b** Panel i Proliferation marker Ki67 staining of prostate at early and late time points of PBP and PKP mice. DLP dorsolateral prostate, AP anterior prostate, VP ventral prostate. Panel ii Quantitative data showed that the intensity of Ki-67 staining was significantly higher in PKP lesions than in PBP lesions. ^***^P < 0.001. **c** Macroscopic appearance and H&E staining of primary and metastatic PCA tumor masses in various organs of PKP mice compared to non-metastatic PBP mice. **d** Representative H&E staining of osteoblastic and osteolytic lesions in PKP mice with skeletal metastasis. **e** Immunostaining of PKP bone metastasis. Osteoblastic marker, TRAcP, and PCA marker CK7 and CK8 were highly expressed in bone metastatic lesions of PKP mice. **f** IHC staining of CK8, PSA, ACPP, and AR in PKP primary and metastatic sites of kidney, pancreas, lymph node, liver, and lung. Scale bar 50 μM

Histologically, PCA in PKP mice resembled human PCA, with regional lymph node invasion, and distant metastasis (Supplementary Table S1). PKP mice had elevated prostate specific antigen (PSA) and prostatic acid phosphatase (ACPP) serum levels, important diagnostic factors for human PCA (Fig. 2a). Molecular characterization revealed increased proliferating cells (Ki67-positive) in PKP glandular epithelium and stroma at early and late time points, unlike PBP mice (Fig. 2b—panels i, ii). PKP mice rapidly developed systemic metastases in lymph node, pancreas, liver, kidney, lung (Fig. 2c), and bone metastasis with osteoblastic and osteolytic lesions (H&E staining and immunohistochemistry (IHC), Fig. 2d, e). Anti-osteoclast-specific enzymes, tartrate-resistant acid phosphatase (TRAcP), and PCA epithelial markers, cytokeratin 7 and cytokeratin 8 (CK7&8) protein confirmed bone resorption and metastases in PKP mice (Fig. 2e). IHC analysis for PCA markers in PKP metastatic lesions recapitulating primary PCA (Fig. 2f).

**Fig. 3:**
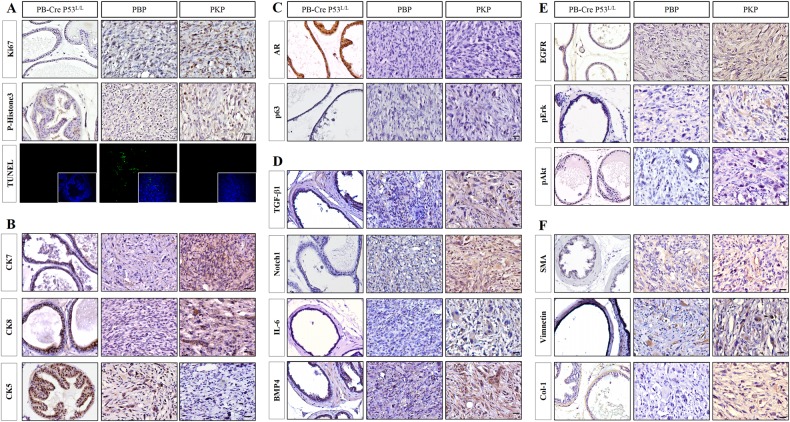
Immunohistochemical analysis of PCA-associated biomarkers, signaling pathways and activated stromal components in control, PKP and PBP prostates. **a** Representative prostate sections analyzed by IHC for Ki67 and p-histone3, with IF analysis for TUNEL assay and distinct levels of protein expression by IHC for **b** CK7, CK8, and CK5; **c** AR and p63; **d** Notch, TGFβ1, IL6, and BMP4; **e** EGFR, pERK, and p-Akt; **f** SMA, vimentin, and type 1 collagen (Col-1) in normal, PBP, and PKP prostates. Scale bar 50 μm

Additional IHC analyses demonstrated alterations in cellular signaling in both PCA models compared to normal murine prostates. Ki67 and p-Histone3 staining showed prominent proliferation in the neoplastic ductal epithelium and stromal fibroblasts in PKP compared to PBP mice (Fig. 3a). PKP mice highly expressed luminal epithelial markers CK7 and CK8 with fewer TUNEL-positive cells than PBP mice (Fig. 3a), while PBP mice stained positive for CK5 but lacked CK7 and CK8 expression, with basal-like/myoepithelial phenotype (Fig. 3b, Supplementary Table S2). Both models stained negative for neuroendocrine marker synaptophysin (Supplementary Fig. S2) and showed substantially decreased androgen receptor (AR) and p63 expression compared to wild type (Fig. 3c, Supplementary Table S2). IL-6, TGF-β, BMP4, and Notch1 were more highly expressed in PKP than PBP and wild-type mice (Fig. 3d, Supplementary Table S2). Since mutant K-Ras may enhance autocrine EGFR ligand expression to trigger multiple signaling pathway in many cancers, we also observed that PKP mice had significantly higher EGFR, p-ERK (p-p44/42), and p-Akt than PBP mice (Fig. 3e, Supplementary Table S2) [28]. Meanwhile, Alcian blue and IHC staining indicated substantially more stromal mucin production in PKP compared with PBP mice (Fig. 3f, Supplementary Fig. S3). Increased expression of extracellular matrix proteins included smooth muscle actin (SMA), type 1 collagen (Col-1), and vimentin in PKP tumors compared to PBP and wild type controls (Fig. 3f), suggesting that Kras^G12D^ activation in prostate epithelium immediately affects surrounding stromal components, demonstrating that our PKP model well recapitulates stromal responses in metastatic human PCA.

### Enhanced tumor cell motility and tumor sphere formation in PCA cells from PKP mice

**Fig. 4:**
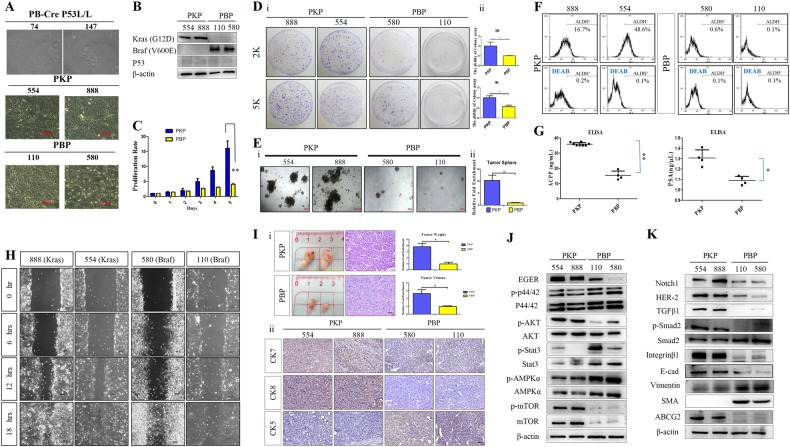
Kras^G12D^ activation combined with p53 loss enhanced PCA cell proliferation, motility, invasiveness, and stemness. **a** Cellular morphology of murine normal prostate gland ducts and PCA cells from PKP and PBP models by phase-contrast light microscope. Scale bar 100 μm. **b** Immunoblot analysis of Kras^G12D^, Braf^V600E^, and p53 protein expression levels in PKP and PBP PCA cells. **c** In vitro cell proliferation assays showing PCA cells from PKP mice grew faster than PBP. Data are mean ± SD, *n* = 3. ^**^P < 0.01. **d** Panel i PKP cells exhibited higher colony forming ability than PBP cells including colony numbers and sizes. Cells (2 and 5 K) seeded on petri dishes were grown for 1 week. Representative images at ×100 magnification. Panel ii Right panel indicates quantitative data. ^*^P < 0.05, ^**^P < 0.01. **e** Murine PCA tumor spheres from PBP and PKP mice. Panel i Photomicrographs (×40 magnification) and panel ii, colony quantification in six random microscopic fields per plate. Error bars, mean ± s.d. of triplicate samples, ^**^P < 0.01. **f** PKP cells expressed higher levels of ALDH activity than PBP cells by Aldefluor flow cytometry-based staining to measure ALDH enzyme activity. Histograms show gated populations without and with ALDH inhibitor, diethylaminobenzaldehyde (DEAB) treatment. **g** ELISA showed significantly higher levels of ACPP and PSA protein in primary PKP cell culture supernatant at 24 h compared to PBP cells (mean ± SE) (*n* = 3 in each group); ^*^P < 0.05, ^**^P < 0.05. **h** In vitro wound scratch assays exhibited higher wound closure ability in PKP than PBP cells. Images captured at 0, 6, 12, and 18 h after wounding. **i** Significantly increased in vivo PKP cell tumorigenesis compared to PBP in xenografts. Panel i Gross xenograft tumor images and xenograft tumor weights and volumes. Mean ± SEM (*n* = 3); ^*^P < 0.05. Panel ii IHC analysis evaluation of CK7, CK8, and CK5 expression in PKP and PBP xenografts. Representative CK7, CK8, and CK5 IHC staining of paraffin-embedded xenograft tumors from SCID mice injected with PKP or PBP PCA cells. **j** Increased p-ERK, p-STAT3, and pAMPKα activation in PBP PCA tumors detected by western blot; with higher EGFR, p-Akt and m-TOR levels detected in PKP PCA. **k** Immunoblots show different expression levels of Notch1, HER2, TGFβ1, p-Smad2/Smad2, EMT markers, and ABCG2 in PKP and PBP PCA cells by western blotting

Cellular morphological examination revealed morphologically distinct cobblestone epithelial cell colonies of normal prostate ductal cells and PCA cells from PKP mice whereas PBP PCA cells were spindle shaped with fibroblastic morphology (Fig. 4a). Protein expression levels of Kras^G12D^, Braf^V600E^, and p53 in primary PKP and PBP PCA cells were confirmed by western blot (Fig. 4b). Kras^G12D^ activation significantly enhanced proliferation compared with PBP cells in methyl tetrazolium (MTT) assays (Fig. 4c). In colony formation and tumor sphere assays, PKP cells showed significantly more anchorage-independent growth than PBP cells (Fig. 4d—panels i, ii,e—panels i, ii) with stronger ALDH1 activity, suggesting more stem cell activity (Fig. 4f). ELISA showed elevated PSA and ACPP protein secretion in PKP PCA cells compared to PBP cells (Fig. 4g).

Next, in vitro motility scratch assays showed significant increases in PKP cell migration compared to PBP cells (Fig. 4h). Kras^G12D^ enhanced PKP PCA cell invasiveness compared to PBP cells by in vitro transwell invasion assay. Primary PKP and PBP cell lines were tested in vivo by subcutaneous (s.c.) xenografts. Kras^G12D^ mutant PCA grew more quickly than PBP cells in xenografts (Fig. 4i—panel i), confirmed by IHC analysis with anti-CK7, CK8, and CK5 antibodies (Fig. 4i—panel ii). To determine the effects of Kras^G12D^ and Barf^V600E^ on EGFR/MEK/ERK and pAkt pathway activation and epithelial–mesenchymal transition (EMT), we examined EGFR/MEK/ERK and Akt the protein levels and phosphorylation status by western blotting. PKP cells exhibited enhanced EGFR expression and increased Akt and m-TOR phosphorylation (Fig. 4j). Conversely, PBP cells showed enhanced phosphorylation of Erk (p44/42), STAT3 and AMPKα. PBP PCA cells acquired a basal-like phenotype with higher vimentin and SMA and lower E-cadherin and Itgaβ1 expression compared to PKP cells, which had higher Notch1, Her2, TGFβ1/Smad expression, and Wnt activity (Fig. 4k, Supplementary Fig. S4A–D).

### K-Ras signaling conferred PCA chemoresistance

We compared the responsiveness of PKP and PBP PCA cells to paclitaxel (Pac) and fluorouracil (5-FU) chemotherapy. Kras^G12D^ expression decreased sensitivity to Pac (5, 10, and 20 μM) and 5-FU (5, 10, and 20 μM), Ras inhibitor manumycin A (5, 10, and 20 μM) and MEK inhibitor PD98059 (5, 10, and 20 μM) compared to PBP cells (Supplementary Fig. S5A). PKP cells also displayed more resistance to the AR antagonist bicalutamide (10 and 20 μM) and tamoxifen (5 and 10 μM) compared to PBP cells (Supplementary Fig. S5A). Conversely, PBP PCA cells were very sensitive to growth inhibition by the BRAF inhibitor vemurafenib (PLX4032) (Supplementary Fig. S5A). Bicalutamide (5 μM) was more effective than spironolactone in vitro in clonogenic assays (Supplementary Fig. S5B). In vitro scratch assays of PKP and PBP cells treated with 5 μM bicalutamide showed suppressed migration (Supplementary Fig. S5C) and transwell invasion assays showed inhibited PKP cell invasiveness after 24 h bicalutamide treatment (Supplementary Fig. S5D). Of note, PKP cell drug resistance may involve increased expression of ABC pump family proteins (Fig. 4k, Supplementary Fig. S6).

### KRAS mutation and P53 loss promote PCA stem-like properties

Next, we defined the molecular circuits mediated by mutant Kras^G12D^ in PCA progression vs. the Braf^V600E^ model by cDNA microarray analysis comparing the gene expression profiles of normal prostatic ducts and early-passage PKP and PBP PCA cells. PKP cells exhibited increased expression of 301 genes and suppressed expression of 195 genes (>fold change 2.0, *P* < 0.05) compared with PBP (Supplementary Fig. S7A). GeneGo pathway analysis identified unique PKP signatures including Development_regulation of EMT, Development_TGFbeta dependent induction of EMT via SMADs, MAPK, RhoA, PI3K, and ILK and Development_Wnt signaling pathways.

Kras^G12D^/p53-loss mediated upregulation of top cancer-associated genes including CD24a (194.8-fold increase, Kras vs. Braf; *P* = 0.003), TMPRSS11E (transmembrane Protease, Serine 11E), Krt7 (keratin 7), Fermt1 (fermitin family homolog 1 (Drosophila)), CDH1 (E-cadherin) (105.7-fold increase; *P* = 0.01), EpCAM (epithelial adhesion molecule, CD326) (89.9-fold increase; *P* = 0.01), WFDC2 (WAP four-disulfide core domain 2, HE4), Claudin 3, 4, and 7 and CD133 (prominin1) (42.1-fold increase; *P* = 0.009) by microarray analysis, All analyzed genes had significantly higher mRNA levels in PKP than in PBP cells verified by real-time quantitative PCR (RT qPCR). (*P* < 0.05, 3-, 1.5-, 21-, 47-, 39-, 1.3-, and 2.7-fold for CD24, TMPRSS11E, CDH1, EPCAM, CD133, FERMT1, and CLDN 3, respectively) (Supplementary Fig. S7B). Some genes were further confirmed at the protein level by western blotting and IHC (Supplementary Fig. S7C, D).

### CD24, CD133, or CD326 knockdown modulates KrasG12D/p53^L/L^ PCA cell growth, motility, invasion, and stemness

**Fig. 5:**
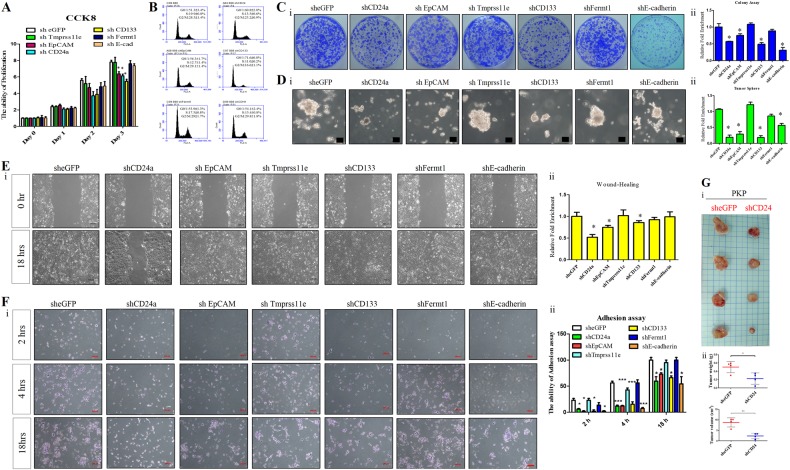
Kras^G12D^-induced CD24, EpCAM, and CD133 modulate cancer stemness traits and promote PCA cell tumorigenesis, migration, and invasion. **a** Cell proliferation assessed as indicated by CCK8 assay. Knockdown of CD24, CD133, and EpCAM significantly reduced primary PKP PCA cell proliferation. ^*^P < 0.05. **b** Cell cycle analysis of shCD24, shCD133, shEpCAM, shE-cadherin, shFERMT1, and mock control PKP cells by flow cytometry. Knockdown of CD24 and CD133 conferred a larger subgroup of G1 cells compared to controls. **c** Clone-forming assays of mock control and indicated knockdown groups. Panel i CD24, CD133, EpCAM, and E-cadherin knockdown in PKP cells suppressed colony formation. Panel ii Quantitative analysis of colony forming assays. Data are mean ± SEM, *N* = 3, ^*^P < 0.05. **d** Sphere-forming assays for shRNA knockdown clones and vector controls revealed that knockdown of CD24, CD133, EpCAM, and E-cadherin suppressed PKP cell self-renewal. Scale bar 100μm. Six random fields (×100) were photographed (i) and sphere numbers counted after two weeks (ii). ^*^P < 0.05. **e** Wound healing migration assays for shRNA knockdown clones and vector controls. Panel i Knockdown of CD24 and EpCAM in PKP cells inhibited migration in vitro. Panel ii Statistical histogram for wound healing assays of indictaed groups. *P* < 0.05. **f** Knockdown of CD24, CD133, EpCAM, and E-cadherin suppressed PKP cell adhesion in vitro. Panel i Representative images from three independent experiments are shown. Panel ii Quantification of adhesion assays that were expressed relative to the eGFP control, setting at 1; ^*^P < 0.05, ^**^P < 0.01. **g** CD24 knockdown inhibited PKP cell xenograft tumor growth. Panel i Gross images of tumors in SCID mice. Panel ii Tumor weights and volumes significantly decreased in mice injected with CD24-knockdown clones compared with controls. mean ± s.e.m., *n* = 4/each groups, ^*^P < 0.05, ^**^P < 0.01

To determine whether upregulated CD24, EpCAM, Tmprss11e, Fermt1, CDH1 (E-cadherin), and CD133 genes sustain PCA cell proliferation, tumor sphere formation, migration, or invasion, we used lentiviral vector-mediated shRNAs to silence CD24, EpCAM, Tmprss11e, Fermt1, E-cadherin, and CD133 mRNA in PKP PCA cells, selecting stable knockdown clones after two weeks’ puromycin (puro) incubation. Western blot confirmed the knockdown efficiency of stable clones (Supplementary Fig. S8). To explore whether gene knockdown with indicated shRNAs influence on tumorigenesis, cell proliferation, cell migration, cell adhesion, and tumor sphere formation assays were performed to compare with sheGFP control cells. Accordingly, as shown in Fig. 5a, the cell proliferation rates in the shCD24, shCD133, shEpCAM, and shE-cadherin of Kras^G12D^/p53^L/L^ PCA cells were significant reduced compared with sheGFP controls by CCK8 assay. The occurrence of growth inhibition was also confirmed by FACS and BrdU assays that showed knockdown of CD24 and CD133 suppress cell cycle progression of PCA cells (Fig. 5b and Supplementary Fig. S9).

Subsequently, colony formation and tumor sphere assays revealed significant decreases in CFUs for CD24, CD133, EpCAM, and E-cadherin shRNA knockdown clones compared to sheGFP controls (*P* < 0.05, Fig. 5c, and d). CD24, CD133, and FERMT1 knockdown significantly decreased wound closure rates compared to sheGFP controls (*P* < 0.05) (Fig. 5e). Suppression of CD24, CD133, EpCAM, and E-cadherin expression reduced PCA cell adhesion (Fig. 5f—panels i, ii). Importantly, when we subjugated KrasG12D/p53 loss PCA cells to four additional rounds of transwell invasion screens, we observed that CD24 is pivotal for maintaining cell migratory activity (Supplementary Fig. S10). Knockdown of endogenous CD24 expression in murine Kras^G12D^/p53 loss PCA cells significantly reduced tumor growth in subcutaneous severe combined immunodeficiency (SCID) xenograft mice (*P* < 0.05, Fig. 5g—panels i, ii).

### CD24, CD133, or CD326 overexpression in PZ-HPV-7 normal human prostate ductal epithelial cells enhances tumorigenesis and migration

**Fig. 6:**
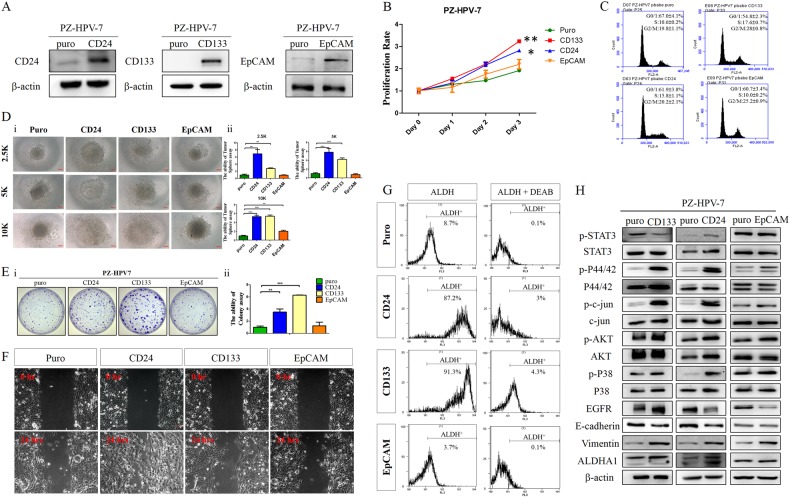
CD24, EpCAM, and CD133 overexpression in human prostate PZ-HPV-7 cells confers CSC properties. **a** Immunoblot analysis confirmed CD24, CD133, and EpCAM overexpression in PZ-HPV-7 cells. **b** MTT assays showed increasing cell growth rates in PZ-HPV-7-CD24 and PZ-HPV-7-CD133 cells compared with puro control. Mean ± SEM, *N* = 3, ^*^P < 0.05, ^**^P < 0.01. **c** Overexpression of CD24, EpCAM, and CD133 protein induced cell accumulation in S phase and decreased the number of cells in G1 phase compared with mock-transfected PZ-HPV-7 cells by FACS analysis. **d** Panel i Spheroid formation of CD133 and CD24 overexpressing PZ-HPV-7 cells displayed larger spheres than controls by hanging drop assays. Panel ii Bar graph quantified the average tumor sphere diameter from the indicated groups. Puro control bars are set at 1, and other bars are relative to puro controls. Data are shown as the mean ± SD (*N* = 3). ^**^P < 0.01, ^***^P < 0.001. **e** Panel i Colony formation assays indicated more colonies in CD24 and CD133 overexpressing PZ-HPV-7 cells stained with 3-(4,5-dimethylthiazol-2-yl)-2,5-diphenyl tetrazolium bromide and photographed. Panel ii Quantified results were expressed relative to the puro control group, setting at 1. Data are expressed as mean ± SD of three independent experiments (^**^P < 0.01, ^***^P < 0.001). **f** Scratch-wound repair at 0 and 24 h postwounding in control and pbabeCD24, CD133, and EpCAM PZ-HPV-7 cells. Overexpression of CD24 in PZ-HPV-7 cells increased in vitro migratory ability. **g** CD24 and CD133 overexpressing PZ-HPV-7 cells expressed higher ALDH activity. ALDH activity was detected in CD24, CD133, EpCAM-PZ-HPV-7 and mock control PZ-HPV-7 cells by Aldefluor-flow cytometry. Baseline fluorescence was established by ALDH inhibitor DEAB. **h** Overexpression of CD24 or CD133 markedly increased phosphorylation of p44/42 (MEK), Akt, and p38 MAPK proteins and induced EMT cascade in PKP cells compared to controls. Representative relative protein levels of p-STAT3, STAT3, EGFR, PTEN, p-c-jun, c-jun, phospho-Akt, (p-Akt), total Akt (Akt), phosphor-p44/42 (p-p44/42), total p44/42, E-cadherin, vimentin and ALDHA1 are shown. β-actin was used as loading control in Western blot analysis

To verify our findings in humans, we stably overexpressed CD24, CD133, and EpCAM by retroviral infection in transformed human prostate epithelial PZ-HPV-7 cells, and confirmed by western blotting following puromycin selection (Fig. 6a). Clones stably overexpressing CD24 and CD133 increased proliferation > 1.5 fold compared to puro control cells (*P* < 0.05) (Fig. 6b). Overexpression of CD24 or CD133 in PZ-HPV-7 cells significantly increased cells in S-phase by FACS analysis (*P* < 0.05; Fig. 6c). In anchorage independent growth assays, overexpression of CD24 and CD133 produced larger tumor spheres than puro controls at 2 weeks (Fig. 6d—panels i, ii). Clonogenic assays showed increased colony-forming ability by CD24 or CD133 overexpressing PZ-HPV-7 cells compared to controls (Fig. 6e—panels i, ii).

CD24 overexpression in human PZ-HPV-7 cells markedly promoted cell migration (*P* < 0.01; Fig. 6f). CD24 and CD133 overexpressing PZ-HPV-7 cells exhibited higher ALDH activity than puro controls by Aldefluor staining (Fig. 6g). Molecular characterization of CD24 and CD133 overexpressing cells displayed Erk, Akt, p38, and c-Jun pathway activation and EMT induction, confirmed by immunoblot analysis (Fig. 6h). We also observed that CD24 modulated Wnt signaling activity in murine and human PCA cells (Supplementary Fig. S4D—panels i, ii). Wnt inhibitor FH535 significantly reduced PCA cell migration in vitro (Supplementary Fig. S4E), clearly showing that CD24-mediated induction of Wnt/β-catenin signaling can enhance PCA cell migration and stemness.

### TGF-α stimulates CD24 expression and CD24 blockade abrogates tumorigenesis and metastasis

**Fig. 7:**
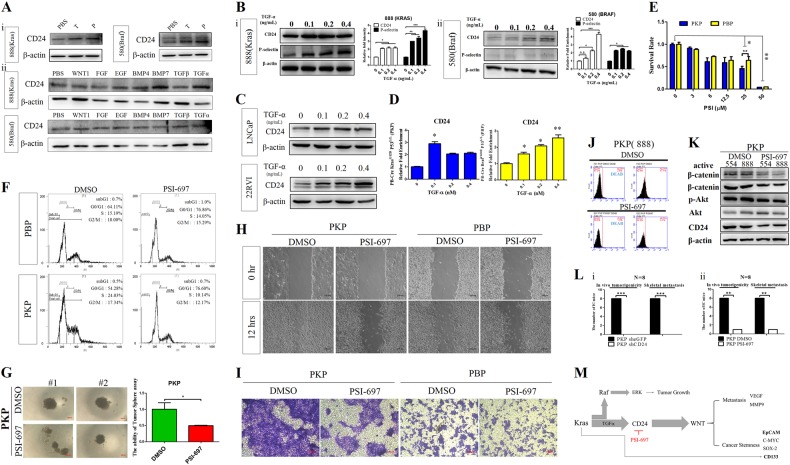
CD24 expression was upregulated by TGFα; targeting CD24/P-selectin suppressed CSC phenotype in PKP cells. **a** Panel i Testosterone (T) and progesterone (P) induced CD24 protein expression in murine PCA cells. Panel ii Immunoblot analysis showing CD24 protein levels after cytokine treatments. CD24 protein expression in murine PBP and PKP cells demonstrated by western blotting after the indicated treatment with Wnt1 (5 ng/ml), FGF(2ng/ml), EGF (1 ng/ml), BMP4 (2 ng/ml), BMP7 (2 ng/ml), TGFα (10 ng/ml), and TGFβ (1 ng/ml) for 18 h. **b** Immunoblot analysis of CD24 and P-selectin protein expression in **a** PKP, **b** PBP cells after treatment with control (×1 PBS) or different TGFα (5 ng/mL) overnight. Values (right panels) were expressed as fold change relative to control (no treatment) and were normalized to actin. Data are means ± SEM from three independent experiments. ^*^P < 0.05, ^**^P < 0.01, ^***^P < 0.001. **c** CD24 protein expression in human LNCaP and 22Rv1 cells with or without TGFα treatment by Western blots. **d** RT qPCR analysis for CD24 expression in **a** murine PKP, **b** PBP, and **c** human LNCaP cells after TGFα treatment. **e** PSI-697 reduced PCA cell in vitro proliferation. Cell proliferation was substantially inhibited in PKP cells treated for 2 days with PSI-697 compared to untreated controls; ^*^P < 0.01, ^**^P < 0.01. **f** FACS analysis showing G1 phase cell cycle arrest and decrease of PCA cells in S phase after PSI-697 treatment. **g** 3D hanging drop formation assays; PSI-679 treatment effectively reduced oncosphere sizes of PKP cells, and the quantitative data of sphere formation assays (right panel). ^**^P < 0.01. **h** In vitro wound scratch assays showing decreased cell migration after PSI-697 treatment under in vitro cell culture conditions. **i** In transwell invasion assays, PSI-697 treatment significantly reduced PKP PCA cell invasion. PSI-697-treated group transmembrane cell numbers were significantly lower than controls at 24 h. **j** PSI-697 treatment of highly metastatic PKP cells decreased their aldehyde dehydrogenase (ALDH) cancer stemness activity. Data indicated a significant reduction of ALDH activity in PKP PSI-697-treated cells at 24 h. (Student’s *t* test, *P* < 0.01). Each experiment was performed three times and representative results were shown. **k**, Western blots revealed that PSI697 treatment reduced protein levels of CD24, active and total β-catenin and phosphorylation levels of Akt. **l** Effects of CD24 silencing (i) or PSI-697 (ii) in PKP PCA cells on inhibitions of cell growth and skeletal metastasis in isogenic mouse model after intracardiac injections. **m** Schematic representation of how Kras signaling upregulates CD24 expression to activate the Wnt signaling pathway, resulting in increasing CSC properties of PCA

Agreeing with previous studies showing that androgen/testosterone can activate CD24 expression in human bladder carcinoma cells, we also found that testosterone increased CD24 expression in our murine PCA models (Fig. 7a—panel i). To investigate other upstream factors regulating CD24 expression, we treated PCA cells with several cytokines and observed changes at the protein level. EGF, BMP7 and TGFα increased CD24 protein levels in murine PCA cells (Fig. 7a—panel ii) and Western blot analysis also revealed increased CD24 and CD24 receptor, P-selectin, protein expression in murine, and human PCA cells after TGFα treatment (Fig. 7b, c). TGFα directly stimulated CD24 gene expression at the transcription level as demonstrated by RT-qPCR (*P* < 0.01) (Fig. 7d). Consistently, TGFα expression was also higher in PKP cells compared to normal murine prostate ductal cells in our cDNA microarray analysis. These results imply that Kras^G12D^/P53 loss-mediated induction of TGFα gene expression might upregulate CD24 expression in our PKP model.

Since increased CD24 expression in murine Kras^G12D^/P53^L/L^ PCA cells directly influenced cell migration, next we assayed whether PSI-697, a bicyclam molecule that antagonizes CD24 binding to its cognate receptor P-selectin, affected PCA cell growth and migration. We found that PSI-697 dose-dependently suppressed PCA cell proliferation (P < 0.01;50 µM) (Fig. 7e), and significantly increased G1 phase cell cycle arrest, reduced the percentage of cells in S phase compared to controls (Fig. 7f). PSI-697 also significantly reduced the number and size of tumor spheroids, implicating the CD24/P-selectin axis in cancer stemness maintenance (Fig. 7g). Furthermore, PSI-697 significantly reduced murine PCA cell migration in scratch assays (Fig. 7h) and cell invasion assays obtained similar effects (Fig. 7i). Meanwhile, our results also demonstrated that PSI-697 significantly reduced ALDH activity in PKP cells (Fig. 7j). Western blot analysis confirmed significant reductions in CD24/P-selectin axis effector proteins after PSI-697 treatment, including decreased levels of p-Akt, active-β-catenin, and total β-catenin compared to PBS treated controls (Fig. 7k). To test the effects of PSI-697 in vivo, shCD24 and eGFP control Kras^G12D^/P53^L/L^ PCA cells were injected intracardially in isogenic graft mice. Suppression of CD24 expression completely abrogated PCA formation and skeletal metastasis (Fig. 7l—panel i, Supplementary Fig. S11). Meanwhile, in vivo PSI-697 treatment also reduced PCA tumorigenesis and metastasis significantly (Fig. 7l—panel ii, Supplementary Fig. S11).

## Discussion

Our two novel mouse models with genetic alterations commonly observed in human PCA recapitulate human pathology and metastatic behaviors. Activation of Kras^G12D^ or BRAF^V600E^ genes combined with P53 deletion induced 100% spontaneous PCA formation. We further compared the contribution of the MAPK and AKT signaling pathways in invasive PCA. We demonstrated that activation of Kras^G12D^ plus p53 deletion caused a widely metastatic PCA phenotype. To understand how mutant Kras with p53 inactivation promotes PCA invasion and metastasis, we conducted systemic analyses of differential gene expression profiles to identify several upregulated cancer-associated genes (CD24a, EPCAM, Tmprss11e, Fermt1, E-cadherin, Claudins and promin1 (CD133)) as potential effectors of mutant Kras^G12D^ compared to normal murine and BRAF^V660E^-driven PCA cells. Among these genes, CD24, EpCAM and CD133 are cell surface adhesion proteins with potential functions relative to CSC activities that have not been fully characterized in PCA.

CD24 was the most prominent CSC marker induced by mutant Kras^G12D^ in our PCA models, followed by EpCAM and CD133. We subjectively focused on CD24, EpCAM, and CD133 based on the facts that they might have more strong effects on modulation of PCA cell proliferation, migration, and invasion. Other studies have confirmed the link between CSC marker expression, tumorigenesis, and metastatic behavior. For instance, CD133 contains five transmembrane regions and two glycosylated extracellular loops with a molecular weight of 97–120 kDa. The CD133 molecule has been detected in human liver, gastric, pancreatic, and prostate tumors, linked to malignancy and invasiveness [29, 30]. Higher CD133 levels in tumor side population (SP) cells than in non-SP cells suggest that CD133 may be a cancer stem cell marker. We previously reported direct interaction between EGFR and CD133 in PDAC and hepatic carcinoma cells, where CD133 activates EGFR signaling [31]. CD133-EGFR interaction may activate MAPK/ERK and PI3K/Akt downstream signaling and CD133 mediated ligand independent EGFR activation may result in increased cancer cell proliferation, adhesion, migration, angiogenesis, and chemoresistance [31]. This study demonstrated that activation of Kras^G12D^ enhanced CD133 expression to support CSC activities in PCA.

Another CSC molecular marker, EpCAM, is a tetrameric transmembrane protein affecting cell–cell adhesion [32]. EpCAM is strongly expressed in somatic stem cells, precursors, and embryonic stem cells. EpCAM (EpC) is a putative CSC marker in liver, pancreatic, breast, and gastric cancer and interacts with cld7 to interfere with cell–cell adhesion, thus enhancing tumor cell migration [33, 34]. In one study, increased expression of EpCAM after TGFβ1 treatment promoted EMT and metastasis [35]. The EpCAM intracellular domain (EpICD) peptide translocates to the cell nucleus following proteolytic cleavage to assemble a transcriptional activation complex with LIM domains, β-catenin, Lef-1, and other transcription factors to activate Wnt target gene expression [36]. This study provides the first evidence linking EpCAM overexpression to oncogenic Kras in PCA. Further studies will focus on the molecular regulation of PCA cancer stemness by EpCAM.

CD24a gene, the most significantly up-regulated CSC marker, encodes a glycogen integrated membrane protein and binds P-selectin to drive endothelial migration and invasion [37]. The CD24/P-selectin binding pathway may affect tumor cell interaction with platelets or endothelial cells in vivo to drive metastasis [38]. CD24 is expressed in lung, kidney, ovarian, and pancreatic cancers [39, 40], and is considered a potent CSC marker whose overexpression indicates lymph node metastasis and poor prognosis [41, 42]. CD24 mRNA levels significantly increase with stage in human PCA in the Cancer Genome Atlas (TCGA) database, associated with positive-lymph node metastasis (Supplementary Fig. S12). CD24^high^ human nasopharyngeal carcinoma cells express stem cell genes (Sox2, Oct4, Nanog, and Bmi-1) with Wnt/β-catenin signaling activation [43]. CD24 is involved in bladder carcinogenesis and metastasis in CD24 deficient mouse model, where androgen and AR promotion of tumorigenesis in male mice depends on CD24 expression [44].

The mechanisms by which PCA induces osteoblastic or osteosclerotic lesions remains elusive [45]. In addition to TGFβ1/BMPs, PTH, RANK, VEGF and FGF, Wnts have also been reported as potential factors mediating PCA skeletal metastasis [46]. Canonical Wnt signaling may contribute to osteoblastic/osteolytic lesions via autocrine and paracrine effects. DKK1 expression suppresses Wnt activity and enhances the osteolytic ability of C4–2B skeletal metastatic PCA [44]. We demonstrated a positive correlation between CD24 expression and the Wnt signaling pathway mediating PCA invasiveness and bone metastasis, while blocking CD24 signaling by shRNA or small molecule inhibitors prevented PCA cell invasion in vitro and metastasis in vivo (Fig. 7i–l). Pharmacological inhibition of CD24/P-selectin signaling with PSI-697 showed promise for treating tumorgenic and metastatic PCA in our models and lay the foundation for further studies interrogating the therapeutic efficacy of PSI-697 for metastatic PCA (Fig. 7m).

In conclusion, Kras^G12D^ and Braf^V600E^ mutations combined with p53 loss rapidly promoted PCA formation. The translational relevance of our findings in PCA is the mechanistic link between mutant Kras and CSC markers, CD24, EpCAM and CD133, which may lead to better prognostic tests, slowing PCA development, and combating metastatic PCA.

## Materials and methods

### Genetically modified mice and mouse genotyping

PB-Cre4, LSL-Kras^G12D^, and p53^Loxp/Loxp^ mice were obtained from the Mouse Models of Human Cancers Consortium (MMHCC). BRaf^F-V600E^ mice were purchased from the Jackson Laboratory (strain B6.129P2(Cg)-Braftm1Mmcm/J). Mutant mice were genotyped by MMHCC and Jackson lab PCR protocols for strains 01XF5, 01XJ6, 01XC2, and 017837. All studies were approved by the Animal Care Committee of the National Sun Yat-Sen University (permit number 10532). Surgery and sacrifice were performed under isoflurane or avertin anesthesia. Blood samples were collected from cardiac puncture. Elements and plasma were separated by centrifugation (3000*g*, 15 min) [47]. Prostate tissue samples were fixed in 10% buffered formalin overnight, washed with ×1 phosphate-buffered saline, transferred to 70% ethanol, paraffin embedded and sectioned for H&E staining.

IHC and immunofluorescence (IF) tissue specimens were isolated following sacrifice, fixed, paraffin embedded, and sectioned as previously described. H&E staining procedures were performed following standard protocols. Alcian blue staining kits was purchased from Scy-Tek Laboratories (Logan, UT, USA) and performed according to the manufacturer’s protocols. Standard procedures for IHC and IF analyses have been described in detail previously [47], and antibodies used in these studies are listed in Supplementary Table S3. The processes of bone decalcification with the Decalcifier II solution were performed according to the manufacturer’s suggested protocol (Leica Biosystems, Buffalo Grove, IL, USA). IHC images of stained slides were captured using an Olympus BX43 upright microscope with a 9 megapixel CCD color digital camera (Olympus Corporation, Tokyo, Japan). Terminal deoxynucleotidyl transferase dUTP nick end labeling (TUNEL) assay was performed according to the manufacturer’s instructions (Promega, WI, USA). Immunofluorescent images were captured using a Delta Vision Personal DV Imaging System (Personal DV Applied Precision, Issaquah, WA, USA).

### Western blot analysis

Western blot analyses were performed using standard protocol as described previously [47]. The primary antibodies used in this study are listed in Supplementary Table S1.

### RNA extraction and microarray analysis

Primary cells grown in culture were scraped and collected by centrifugation, and total RNA was subsequently isolated using the RNeasy Mini Kit (Qiagen Inc., Valencia, CA, USA; P/N 74104). RNA quantity and purity were assessed via a 260/280 nm ratio using a Nanodrop ND-1000 machine (Labtech International Ltd., Rigmer, UK). For all sample, 300 ng of total RNA was amplified and labeled using the GeneChip WT Sense Target Labeling and Control Reagents (900652) for Expression Analysis.

### Complementary DNA microarray analysis

Hybridization of labeled samples was performed against the Affymetrix GeneChip MoGene 1.0 ST array for 17 h at 45 °C and 60 r.p.m. Arrays were subsequently washed (Affymetrix Fluidics Station 450, Santa Clara, CA, USA) and stained with streptavidin–phycoerythrin (GeneChip Hybridization, Wash, and Stain Kit, Affymetrix, Santa Clara, CA, USA; 900720), and scanned on an Affymetrix GeneChip Scanner 3000. The resulting data were analyzed using Expression Console software (Affymetrix) and Transcriptome Analysis Console software (Affymetrix) with default RMA parameters. Differentially regulated genes between samples were identified using >2.0-fold change and *P* value < 0.05

### GeneGo analysis

Differentially regulated gene lists were uploaded from a Microsoft Excel spreadsheet onto Metacore 6.13 software (GeneGo pathways analysis; http://www.genego.com). GeneGo recognizes the Affymetrix identifiers and maps them to the MetaCore data analysis suite, generating maps to describe common pathways or molecular connections between samples on the list. Graphical representations of the molecular relationships between genes were generated using the GeneGo pathway analysis, based upon processes showing significant (*P* < 0.05) association.

### RT qPCR analysis

RT–qPCR was carried out using the Biorad CFX Connect (Bio-Rad Laboratories, CA, USA), and reactions and protocols were followed as previously described [47]. The primers for RT qPCR are listed in Supplementary Table S4.

### Cell proliferation assay

Cell proliferation assays were performed using standard MTT-based cell growth assay as described previously [47]. A Cell Counting Kit-8 (CCK-8) assay (Dojindo Inc., Kumamoto, Japan) was performed following manufacturer’s protocol. BrdU proliferation assay was performed using a kit purchased from Cell Signaling Technology (Danvers, MA, USA).

### Murine primary prostate cancer cell culture, cytokines, and inhibitors

The mouse primary prostate cancer cells were cultured in RPMI-1640 medium supplemented with 10% fetal bovine serum, nonessential amino acids, 100 units/ml penicillin and 100 μg/ml streptomycin at 37 °C in a 5% CO_2_ incubator. Primary mouse prostate glandular and PCA cells were maintained for <six passages and histopathologically characterized through SCID mice xenograft studies before performing microarray expression profile analyses. Spheroids were created using Perfecta3D^®^ Hanging Drop Plates (Sigma Aldrich, St. Louis, MO, USA). Spheroids of cells (2 × 10^3^ cells) were prepared as described above. Cells were treated with the following compounds: Paclitaxel (T7042), Fluorouracil (5-FU, F8423), Bicalutamide (CDX, B9061), Spironolactone (S3378), Tamoxifen (T5648), Manumycin A, (M6418), Gefitinib (SML1657), PD98059 (P215), IL-6 (I9646), EGF (E9644), FGF(F5392), Testosterone (T6147), Progesterone (P8783), WNT-1(SRP4754), and TGFα (T7924) were obtained from Sigma Aldrich (St. Louis, MO, USA). TGF-β (240-B), BMP4 (314-BP) and BMP7 (3008-WN-025) were obtained from R&D Systems (Minneapolis, MN, USA). Vemurafenib (PLX4032) was purchased from Selleck Chemicals, and PSI-697 (HY-15526) was purchased from Medchem Express.

### Human prostate cell culture

PZ-HPV-7 cells obtained from Dr. Chuu laboratory in NHRI (Miaoli, Taiwan) were grown in keratinocyte serum-free medium (Gibco, USA) supplemented with 5 ng/mL human recombinant EGF and 50 ng/mL bovine pituitary extract [48]. LNCaP clone FGC (LNCaP) and 22Rv1 cells purchased from the American Type Culture Collection/BCRC (Bioresource Collection and Research Center, Taiwan) were maintained in RPMI-1640 supplemented with 10% fetal bovine serum and 1% antibiotic penicillin and streptomycin. These cells have performed STR PCR profiles at TopGen Biotechnology Co., (Kaohsiung, Taiwan). All cell cultures were mycoplasma-free by PCR, validated by Nautia Gene (Taipei, Taiwan).

### Wound-healing assay

Cells were pretreated with 0.02% (0.2 mg/mL) Mitomycin C for 2 h and wounded by removing a 300–500 mm wide strip of cells across the well with a standard 200 mL yellow tip. Wounded monolayers were washed twice with phosphate-buffered saline to remove non-adherent cells. The cells were cultured in low FBS media and incubated for predetermined times to monitor wound closing. Wound closure was recorded by phase-contrast microscopy as described previously [47].

### Soft agar colony formation assay

Aliquots of cells (10 × 10^3^) were suspended in 1 mL of RPMI-1640 medium with 10% FBS containing 0.3% agarose and plated in triplicate on a firm 0.6% agarose base in 60 mm tissue culture dishes. After 14 days, the cells were washed with PBS and fixed with methanol and 0.1% crystal violet. The colonies were photographed and manually counted.

### Retroviral production and infection of target cells

Retrovirus was generated by cotransfection of the pBabe empty vector, pBabe puro-CD24 (Addgene), pBabe puro-EpCAM or pBabe puro-CD133 with pVSV-G (envelope) and packaging pGAG-POL plasmids in 293 T cells. Retrovirus production and infection were conducted as described previously [47].

### AldeRed ALDH detection assay

Aldefluor assay was performed using the AldeRed ALDH Detection Assay (Merck) according to manufacturer’s instruction. Flow cytometry was performed using BD ACCURI C6 flow cytometer (BD Biosciences, NJ, USA)

### ELISA

Plasma or conditional medium PSA and ACPP concentrations were determined by ELISA kits (mouse PSA and ACPP kit, MyBiosource, CA, USA) following the manufacturer’s protocol.

### Lentivirus production and shRNA for gene knockdown

The plasmids required for shRNA lentivirus production were purchased from the National RNAi Core Facility, Academia Sinica, Taiwan. The pLKO.1-shRNA vectors used for knockdown were as follows; TRCN0000077028 (CD24a), TRCN00- 00111222 (EpCAM), TRCN0000115319 (PROM1/CD133), TRCN0000173934 (FERMT1), TRCN0000032331 (TMPRSS11E) and TRCN0000042579 (CDH1/ E-cadherin). The pLKO.1-shEGFP control plasmid was TRCN0000072190 (EGFP). Lentivirus production and infection were performed following the previously described protocol [47].

### Magnetic resonance imaging

Mice were anesthetized with 1–2 isolfurance/air, and body temperature was maintained by air conditioning through the bore of the magnet ring. Magnetic resonance imaging scans were performed using a 3 T MRI scanner (GE, HDXt Sigma; GE, Milwaukee, WI, USA) with a high-resolution animal coil (3.0 cm diameter). Mice were placed supine in the coil, taped below the thoracic cavity on the bed to reduce respiratory motion. T2-weighted images were acquired using a fast spin echo multi-slices sequence with TR/TE 2000/63.23 ms for coronal section and 5083/46.7 ms for axial section, 16 echo trains, 4 averages, 2 dummy scans, field of view = 8 × 4.8 cm^3^, for coronal section and 6 × 6 cm^2^ for axial section, matrix size = 256 × 192, slice thickness = 2 mm, and number of slices = 20 contiguous. Scans were captured every 10 min until the 90-min mark was reached. A glass cylinder of pure water was positioned adjacent to each mouse as a standard reference.

### Intracardiac injection of metastatic tumor model

The mixed background B6; 129 male mice (*n* = 6 each group; 20–22 g) were maintained in a well-controlled pathogen-free environment. Intracardiac (ic) injection of prostate cancer cells was performed to allow murine PCA cells (shCD24 vs. eGFP control) to disseminate into multiple organs including bone. Briefly, mice (>6 weeks old) were anesthetized with Tribromoethanol and murine PCA cells (2 × 10^4^ cells per mouse) were injected into the left heart ventricle of isogenic recipient male mice [49]. The mice were sacrificed after day 30 and all metastatic organs were collected and embedded in paraffin, sectioned, and stained with H&E as described above.

### PSI-697 in vivo treatment study

In this experiment, murine PCA cells were injected into the heart’s left ventricle of 8-week-old isogenic mouse as described above. Two days after the PCA cell injection, mice were randomly separated to two groups with >4 mice per group. For in vivo treatment, PSI-697 (10 mg) was dissolved in 2.7186 ml of dimethyl sulfoxide (DMSO) solution to a final concentration of 10 mM before use [50]. In the treatment group, each experimental mouse was administered 50 mg/kg PSI-697 twice weekly via intraperitoneal injections for 3 weeks and compared to DMSO treated control group (*N* > 4 per group). At the end of the experiment, mice were sacrificed by anesthetizing with avertin, and all metastatic organs were collected and followed by H&E staining for standard histological examination.

### Statistical analysis

All experiments were repeated at least three times. One representative experiment is shown. RT qPCR and cell proliferation assays are displayed as one representative experiment of three independent experiments, mean ± s.e.m. Data measured on continuous scale were analyzed using Student’s *t* test and categorical data were subjected to *χ*^2^ test. *P* value < 0.05 was considered significant.

### Accession codes

Microarray data are available in the Gene Expression Omnibus (GEO), accession number GSE100919.

## Electronic supplementary material

Supplementary figure 1-12

Supplementary informations

Supplementary Table S1

Supplementary Table S2

Supplementary Table S3

Supplementary Table S4
