# Supplementary material for: Mutant Kras-induced upregulation of CD24 enhances prostate cancer stemness and bone metastasis
Source: Oncogene. 2018 Nov 22;38(12):2005–19. doi: 10.1038/s41388-018-0575-7 (PMC6484710; doi:10.1038/s41388-018-0575-7)
Supplement: Supplementary file 2 — Supplementary informations [file 41388_2018_575_MOESM2_ESM.pdf]

Supplementary information.

Supplementary Table S1. Comparison of incidence percentages of organs involvement in metastatic prostate cancer derived from PB-Cre LSL-KrasG12D p53L/L (PKP) and PB-Cre BRAFV600E p53L/L (PBP) mice.

Supplementary Table S2. Semi-quantitative evaluation of IHC staining of indicated proteins in the prostates tissues of wild type (PP), PBP and PKP mice.

Supplementary Table S3. List of the primary antibodies used in this study, and information on working dilutions of antibodies in Western blotting (WB), immunohistochemistry (IHC) and immunofluorescence (IF).

Supplementary Table S4. List of the primers used in this study.

Supplementary Figure Legends

Supplementary Figure S1. Conditional activation of Kras^{G12D} or BRAF^{V600E} alone in developing prostate has no detectable effects on prostate development and does

not initiate any PCA formation. The figures present low (100-200x)- and high-(400x) magnification images of H&E stained histological sections of prostate tissue from 20- and 30-week-old PB-CreBRAF^{V600E} and PB-CreKras^{G12D} compound mice. Morphological and histological analysis revealed that activation of the Kras^{G12D} or BRAF^{V600E} gene in the murine prostate did not affect prostate maturation and function or play a role in prostate tumorigenesis. Scale bars as shown on individual images.

Supplementary Figure S2. Immunohistochemistry analysis revealed no neuroendocrine marker synaptophysin staining e in PCA lesions derived from PKP and PBP mice. Representative IHC images of PCA tissues derived from PKP and PBP mice using anti-synaptophysin antibody. Scale bar was 100 μm. The mouse brain section was stained by immunohistochemistry with anti- synaptophysin antibody as a positive control.

Supplementary Figure S3. Mucin chemical and IHC staining of PCA derived from PKP and PBP mice. A, Alcian Blue stained in histologic sections of PCA from PB-Cre; P53$^{L/L}$, PKP and PBP mice. B, IHC staining for Mucin in the sections from paraffin-embedded PCA derived from wild type, PB-Cre; P53$^{L/L}$, PKP and PBP mice. Images taken at 200x magnification are shown.

Supplementary Figure S4. Wnt signaling pathway mediates Kras^{G12D} –induced CD24 expression to enhance in vitro cell migratory ability of PCA cells. A, RT qPCR analysis demonstrates that Wnt associated gene expression is altered in PKP PCA cells compared to PCA cells from PBP mice. Graphical representation demonstrating that expression of MMP9, SOX2, C-MYC and LRP5 in PKP groups were significant increase as compared to PBP groups, whereas the expression of DKK2 and THBS were reduced in PKP groups as compared with PBP ones (n=4; *P<0.05, **P<0.001; error bars show s.e.m.). B, Western blot indicated increased Wnt pathway associated protein expression in the PCA cells from PKP mice compared to that of PBP group. C, IHC staining for active-β-catenin (ABC), β-catenin, c-Myc and LRP5 in prostate sections of PB-Cre p53$^{L/L}$, PBP and PKP mice. (magnification, 200×). D, i, the expression of β-catenin, ABC, LRP5 and Cyclin D in shCD24 PKP cells significantly decreased when compared to the sheGFP control PKP cells as demonstrated by western blot analysis. **ii** CD24 overexpressing PZ-HPV-7 cells increase the expression levels of ABC, LRP5, COX-2, Cyclin D and Bcl-2 protein compared to HPV-P7 mock controls were detected by Western blot in which β-actin served as internal control. E, Wnt inhibitor FH535 treatment (2μg/ml) significantly inhibits migratory ability of murine PCA cells as assessed by in vitro wound healing assays.

Supplementary Figure S5. Mutant KRas^{G12D} confers to increase chemoresistant and bicalutamide treatment suppressed PCA cell colony formation and migration. A, Kras exhibits chemo resisitance phenotypes for Pac, 5-FU and bicalutamide treatment as demonstrated by in vitro MTT cell proliferation assays. Cells were treated with or without (control) or with Pac, 5 FU, bicalutamide, PD98059, tamoxifen, manumycin A, gefitinib and PLX4032. After 48 hours, cell growth rate was measured by MTT assay and results were reported as a percentage relative to untreated cells. B, colony formation assay showed that bicalutamide markedly suppressed cell colony formation abilities of PKP and PBP cell lines, whereas, spironolactone did not show any inhibition on colony formation. Clonogenic assay of PKP and PBP PCA cells treated with DMSO, bicalutamide (5μM) or spironolactone (5μM) for 7 days as detailed; photographs of petri dishes in a representative experiment are shown. C, wound healing assays showed that bicalutamide remarkably reduced cell motility in PCA cells. DMSO or bicalutamide (5μM) treatment on wound healing was assayed by wounding the monolayer cells using a sterilized pipette tip. Monolayer cells were photographed at 0, 6, 12 and 18 hrs. Magnification, ×40. Representative results of three different experiments. $P<0.01$. D, transwell invasion assays depicting the reduced invasive ability of murine PCA cells after bicalutamide (5μM) treatment. The images showed

that the ability of invasion was significantly decreased in bicalutamide treated cells as compare to controls.

Supplementary Figure S6. Kras^{G12D} stimulates ABCB1, ABCC1 and ABCG2 gene expression in murine PCA cells. RT-qPCR analysis for ABCC1 and ABCB1 expression in PCA cells from PKP model compared to PCA cells from PBP mice. Results are presented as ratios of ABCC1, ABCB1 and ABCG2 mRNA normalized to internal GAPDH. Representative results of three different experiments. $P<0.05$.

Supplementary Figure S7. cDNA microarray analysis of primary murine PCA cells from PKP and PBP mice. A, heat- map presentation of gene profiling of normal primary prostate ductal gland cells and primary PCA cells established from 2 pairs of normal wild type mice, PKP and PBP mice showing genes with significantly increased (red), intermediate (black) and decreased (green) expression levels. The numerical values give the actual values on a log 2 scale associated with each color. Microarray data details are available at the National Center for Biotechnology Information (GSE 100919). B, qRT-PCR analysis confirmed the most significant gene set induced by the alterations of PKP that showed changes in the microarray analysis as compared to PBP groups. The relative gene expression was normalized to GAPDH expression and

compared with the PBP groups. Data represent the means ± SD of triplicate samples. *$p < 0.05$; **$p < 0.01$, t-test. C, western blot analysis confirmed protein expression levels of CD24, EpCAM, CD133, Fmprss11e, Fermt1, CK7, CK8, CK5, Mal2, TGF-α and ACTA2, from primary PCA cells derived from PKP and PBP mice. β-actin serves as a loading control. D, IHC analysis verified the increased expression of CD24, EpCAM, CD133, CK7 and CK8 in PCA from PKP mice as compared to that of PBP groups. Scale bar:100μm

Supplementary Figure S8. Validation of each shRNA clones knockdown efficiency in primary PCA cells from PKP mice by western blot. Western blot confirmed that after stable selection (1μg/ml puromycin) of shRNA transfection clones with the indicated shRNA knockdown vectors, the expression of target knockdown gene was significantly down regulated, respectively. β-actin was used as the loading control.

Supplementary Figure S9. Cell proliferation was analyzed by BrdU cell proliferation assay. Knockdown of CD24, CD133 significantly suppressed PKP cells proliferation. DNA synthesis was measured by BrdU incorporation assay at 72 hrs. Fold change in DNA synthesis was calculated by comparing BrdU signals of indicated shRNA knockdown stable cell clones to that of the shRNA control, to which a value of

100 was assigned. Statistical significance was evaluated by the Mann-Whitney U test. *; $P<0.05$.

Supplementary Figure S10. The invasive property of PKP cells is associated with the increase of CD24 expression. A, the highly invasive PKP cells were subcloned and determined by 4 additional enrich rounds of the transwell assay. And bottom (migrated) cells were fixed and stained with crystal violet. The representative fields were photographed at ×10 magnification. 4 enrich rounds of the Transwell invasion assay were performed to obtain highly invasive PKP clones. After several rounds of enrichment, highly invasive clones are recovered and molecular characterized by western blot analysis and repeated three times. Cells were stained with crystal violet and photographed. B, immunoblots of CD24 protein and other related CSC proteins after 4 enrich rounds of the transwell assays in PKP cells. Significantly increased expression of CD24, MMP9 and FERMT1 were shown, while there is decreased on the expression of EpCAM, E-cadherin and CD133.

Supplementary Figure S11. Knockdown of CD24 or inhibition of CD24/P-selectin signaling suppresses PCA formation and bone metastasis in metastatic bone tumor model in mice. A, representative autopsy images of the abdomen of intracardiac (i.c)

inoculation of eGFP, shCD24 PCA cells as well as the isogenic tumor graft model intraperitoneally treated with PSI-697 or vehicle (DMSO) for 3 weeks showing the significant reduction of PCA formation and bone metastasis in the shCD24 PCA cells and PSI-697 treated isogenic tumor graft models comparing with the eGFP control and DMSO groups. B, macroscopic appearance and H&E histological analysis of murine PCA formation and bone metastasis in cardiac injection-induced metastatic bone tumor model in mice. T, prostate cancer tumor. Scale bar, 50μm.

Supplementary Figure S12. CD24 overexpression in human PCA. A, in stage 3+4 PCA, CD24 mRNA levels were significantly increased compared with the stage 1 and stage 2 tissues from the TCGA database. *P<0.001 vs. stage 1 (N= 400) by Mann–Whitney *t* test. B, analyzing results from TCGA database showed that CD24 overexpression correlates with lymph node metastasis (N1) in PCA, *$P < 0.05$ (N=417).
