# Supplementary material for: Mutant Kras-induced upregulation of CD24 enhances prostate cancer stemness and bone metastasis
Source: Oncogene. 2018 Nov 22;38(12):2005–19. doi: 10.1038/s41388-018-0575-7 (PMC6484710; doi:10.1038/s41388-018-0575-7)
Supplement: Supplementary file 3 — Supplementary Table S1 [file 41388_2018_575_MOESM3_ESM.pdf]

Supplementary Table S1. Comparison of incidence percentages of organs involvement in metastatic Prostate Cancer derived from PB-Cre LSL-Kras^{G12D} p53$^{L/L}$ (PKP) and PB-Cre BRAF^{V600E} p53$^{L/L}$ (PBP) mice.

	Metastasis	Total	Percent	
Liver				P=0.0502
PBP	0	24	0%	
PKP	5	25	20%	
Lymph node				P=1.0000
PBP	5	24	20.8%	
PKP	5	25	16%	
Pancreas				P=0.0006
PBP	0	24	0%	
PKP	10	25	40%	
Lung				P=0.0046
PBP	1	24	4.1%	
PKP	10	25	40%	
Kidney				P=0.0374
PBP	2	24	8.33%	
PKP	9	25	36%	
Bone				P=0.0223
PBP	0	24	0%	
PKP	6	25	24%	

Categorized parameters were compared using Fisher's exact test
