# Supplementary material for: Mutant Kras-induced upregulation of CD24 enhances prostate cancer stemness and bone metastasis
Source: Oncogene. 2018 Nov 22;38(12):2005–19. doi: 10.1038/s41388-018-0575-7 (PMC6484710; doi:10.1038/s41388-018-0575-7)
Supplement: Supplementary file 4 — Supplementary Table S2 [file 41388_2018_575_MOESM4_ESM.pdf]

Supplementary Table S2. Semi-quantitative evaluation of IHC staining of indicated proteins in the prostates tissues of wild type (PP), PBP and PKP mice.

Antibody	PP	PBP	PKP
Ki67	- /+	++	+++
p-Histone H3	-/+	+	+++
TUNEL	-	+	-
CK7	+	+	+++
CK8	+	+	++
CK5	++	+++	-
AR	+++	-/+	-/+
P63	+	-	-
BMP4	+	+	++
EGFR	-	+	++
p-AKT	+	+	++
p-P44/42	-	+	++
TGF-β1	+	+	+++
IL-6	-	+	++
Notch1	+	++	+++
Vimentin	+	++	++
Collagen I	+	+	+++
SMA	-	++	++

Formalin-fixed paraffin sections. Scores are based on the extent and intensity of staining (- negative; -/+, fewer than 5 positive cells/section; +, weak reactivity; ++, moderate/discontinuous reactivity; +++, high/continuous reactivity).
