# Supplementary material for: Mutant Kras-induced upregulation of CD24 enhances prostate cancer stemness and bone metastasis
Source: Oncogene. 2018 Nov 22;38(12):2005–19. doi: 10.1038/s41388-018-0575-7 (PMC6484710; doi:10.1038/s41388-018-0575-7)
Supplement: Supplementary file 5 — Supplementary Table S3 [file 41388_2018_575_MOESM5_ESM.pdf]

Supplementary Table S3. List of the primary antibodies used in this study, and information on working dilutions of antibodies in Western blotting (WB), immunohistochemistry (IHC) and immunofluorescence (IF).

Antigen	Species	Source	Catalog#	Dilution	Application
Ki67	Rabbit	Abcam	ab16667	1:100	IHC
p-Histon H3	Rabbit	Santa Cruz Biotechnology	sc-8656-R	1:100	IHC
AR	Rabbit	Santa Cruz Biotechnology	sc-816	1:200	IHC and WB
P63	Mouse	Abcam	ab735	1:200	IHC
BMP4	Mouse	Santa Cruz Biotechnology	sc-12721	1:100	IHC
EGFR	Rabbit	Cell Signaling Technology	#9922	1:100	WB and IHC
p-Akt	Rabbit	Cell Signaling Technology	#9271	1:100	IHC and WB
AKT	Rabbit	Cell Signaling Technology	#4691S	1:2000	WB
p-p44/42	Rabbit	Cell Signaling Technology	#9100	1:200	WB and IHC
P44/42	Rabbit	Cell Signaling Technology	#4	1:2000	WB
SMA	Rabbit	Abcam	ab5694	1:100	WB and IHC
Vimentin	Mouse	Santa Cruz Biotechnology	sc-32322	1:100	WB and IHC
Collagen type I	Goat	Santa Cruz Biotechnology	sc-25974	1:100	IHC
Notch1	Goat	Santa Cruz Biotechnology	sc-6014	1:100	WB and IHC
TGF-beta1	Rabbit	Abcam	ab66043	1:200	IHC
IL-6	Rabbit	Santa Cruz Biotechnology	sc-7920	1:100	IHC
Cytokeratin 7	Mouse	Santa Cruz Biotechnology	sc-23876	1:200	IHC and WB
keratin 8	Rabbit	ABGent	P05787	1:1000	IHC and WB
Cytokeratin 5	Rabbit	Biorbyt	orb10408	1:100	IHC and WB
Kras	Mouse	Santa Cruz Biotechnology	sc-30	1:200	WB
Raf-B	Mouse	Santa Cruz Biotechnology	sc-5284	1:200	WB

P53	Rabbit	Cell Signaling Technology	#2527	1:1000	WB
p-STAT3	Rabbit	Cell Signaling Technology	#9145	1:1000	WB
STAT3	Mouse	Cell Signaling Technology	#9139	1:1000	WB
p-AMPK α	Rabbit	Cell Signaling Technology	#2535	1:1000	WB
AMPKα	Rabbit	Cell Signaling Technology	#2603	1:1000	WB
p-mTOR	Rabbit	Cell Signaling Technology	#2971	1:1000	WB
mTOR	Rabbit	Cell Signaling Technology	#2972	1:1000	WB
Her-2	Rabbit	Cell Signaling Technology	#4290P	1:1000	WB
Integrin β 1	Rabbit	Santa Cruz Biotechnology	sc-8978	1:200	WB
E-cadherin	Rabbit	Cell Signaling Technology	#4065	1:1000	WB
ABCG2	Mouse	Santa Cruz Biotechnology	sc-58222	1:200	IHC and WB
p-Smad2	Rabbit	Cell Signaling Technology	#3140	1:1000	WB
Smad2	Rabbit	Cell Signaling Technology	#3122	1:2000	WB
β-actin	Mouse	Sigma	A5316	1:4000	WB
CD24	Rat	Abcam	ab64064	1:500	IHC and WB
EpCAM	Mouse	Santa Cruz Biotechnology	sc-66020	1:200	IHC and WB
CD133	Rabbit	Abnova	600-190	1:200	IHC and WB
Fermt1	Rabbit	Abcam	ab105360	1:500	IHC and WB
Tmprss11e	Goat	Santa Cruz Biotechnology	sc-169638	1:200	WB
Mal2	Rabbit	Santa Cruz Biotechnology	sc-87993	1:200	IHC and WB
ACAT2	Mouse	Santa Cruz Biotechnology	sc-32251	1:200	WB
CD44	Rabbit	Santa Cruz Biotechnology	sc-7946	1:200	IHC and WB
p-P38	Rabbit	Cell Signaling Technology	#9215	1:1000	WB
P38	Rabbit	Cell Signaling Technology	#9213	1:2000	WB

ALDH	Rabbit	Santa Cruz Biotechnology	sc-50385	1:100	WB
p-c-Jun	Rabbit	Cell Signaling Technology	#9261	1:1000	WB
c-Jun	Rabbit	Cell Signaling Technology	#9165	1:1000	WB
β-catenin	Mouse	Santa Cruz Biotechnology	sc-7963	1:50	IHC and WB
Active-β-catenin (ABC)	Mouse	Merck Millipore	#05-665	1:100	IHC
c-myc	Rabbit	Santa Cruz Biotechnology	sc-788	1:200	WB
TRAcP	Mouse	ScyTek	RA0422-c.5	1:100	IHC
Nkx-3.1	Goat	Santa Cruz Biotechnology	sc-15022	1:200	IHC and WB
p-GSK3 *β*	Rabbit	Cell Signaling Technology	#9336	1:1000	WB
GSK3 *β*	Rabbit	Cell Signaling Technology	#9315	1:1000	WB
Mucin2	Rabbit	Santa Cruz Biotechnology	sc-15334	1:100	IHC
PSA	Rabbit	Abcam	ab53774	1:200	IHC and WB
Synaptophysin	Rabbit	Abcam	Ab32127	1:400	IHC and WB
