# Supplementary material for: Mutant Kras-induced upregulation of CD24 enhances prostate cancer stemness and bone metastasis
Source: Oncogene. 2018 Nov 22;38(12):2005–19. doi: 10.1038/s41388-018-0575-7 (PMC6484710; doi:10.1038/s41388-018-0575-7)
Supplement: Supplementary file 6 — Supplementary Table S4 [file 41388_2018_575_MOESM6_ESM.pdf]

Supplementary Table S4. List of the primers used in this study.

Gene name	Forward Sequence 5' to 3'	Reverse Sequence 5' to 3'
CDH1	GGTCATCAGTGTGCTCACCTCT	GCTGTTGTGCTCAAGCCTTCAC
DCN	ACTCTCCAGGAACTTCGTGTCC	AGTCCCTGGAAGGCTCCGTTTT
ACTA1	ACCATCGGCAATGAGCGTTTCC	GCTGTTGTAGGTGGTCTCATGG
BGN	TGAACCAGGAGCCTTTGATGGC	GTCCTCCAACTCAATAGCCTGG
PROM1(CD133)	CTGCGATAGCATCAGACCAAGC	CTTTTGACGAGGCTCTCCAGATC
CD24A	ACATCTGTTGCACCGTTTCCCG	CAGGAGACCAGCTGTGGACTG
EPCAM	GAGTCCGAAGAACCGACAAGGA	GATGTGAACGCCTCTTGAAGCG
CK7	CGGAGATGAACCGCTCTATCCA	CATGAGCATCCTTGATTGCCAGC
PTHLH	GGCGTTCGGTGGAGGGGCTT	CAGATGGTGGAGGAAGAAACGG
MMP9	GCTGACTACGATAAGGACGGCA	TAGTGGTGCAGGCAGAGTAGGA
RANKL	GTGAAGACACACTACCTGACTCC	GCCACATCCAACCATGAGCCTT
OPG	GTGAAGACACACTACCTGACTCC	GCCACATCCAACCATGAGCCTT
OPN	CTGGAGGTCTTCTACAACTCGG	GTGAACTCCAGCAGGGAATGAG
Tgfa	CAGGCTCTGGAGAACAGCACAT	GACACATGCTGGCTTCTCTTCC
Dkk2	CGGCATAGAGATCGCAACCATG	GCAGTCTGATGACCGTAGGCAT
THBS2	GTATGGAGGGAAGGACTGTGTC	ACTTGGCTCCAGGAAAACACGG
LRP5	CCTCACCATTGATTATGCCGACC	GATCGTCAGCTATCACCATGCG
WNT1	CGAGAGTGCAAATGGCAATTCCG	GATGAACGCTGTTTCTCGGCAG
ACTA2	TGCTGACAGAGGCACCACTGAA	CAGTTGTACGTCCAGAGGCATAG
C-MYC	TGCTGGACACGCTGACGAAAGT	GCCAATTCTAGGCGAAGCAGCT
